# Catalase, superoxide dismutase and ascorbate-glutathione cycle enzymes confer drought tolerance of *Amaranthus tricolor*

**DOI:** 10.1038/s41598-018-34944-0

**Published:** 2018-11-07

**Authors:** Umakanta Sarker, Shinya Oba

**Affiliations:** 1 The United Graduate School of Agricultural Science, Laboratory of Field Science, Faculty of Applied Biological Sciences, Gifu University, Yanagido 1-1, Gifu, Japan; 2 Department of Genetics and Plant Breeding, Faculty of Agriculture, Bangabandhu Sheikh Mujibur Rahman Agricultural University, Gazipur, 1706 Bangladesh

## Abstract

The study was performed to explore physiological, non-enzymatic and enzymatic detoxification pathways of reactive oxygen species (ROS) in tolerance of *Amaranthus tricolor* under drought stress. The tolerant genotype VA13 exhibited lower reduction in growth, photosynthetic pigments, relative water content (RWC) and negligible increment in electrolyte leakage (EL), lower increment in proline, guaiacol peroxidase (GPOX) activity compared to sensitive genotype VA15. This genotype also had higher catalase (CAT), superoxide dismutase (SOD), remarkable and dramatic increment in ascorbate-glutathione content, ascorbate-glutathione redox and ascorbate-glutathione cycle enzymes activity compared to sensitive genotype VA15. The negligible increment of ascorbate-glutathione content, ascorbate-glutathione redox and ascorbate-glutathione cycle enzymes activities and dramatic increment in malondialdehyde (MDA), hydrogen peroxide (H_2_O_2_) and EL were observed in the sensitive genotype VA15. SOD contributed superoxide radical dismutation and CAT contributed H_2_O_2_ detoxification in both sensitive and tolerant varieties, however, these had a great contribution in the tolerant variety. Conversely, proline and GPOX accumulation were higher in the sensitive variety compared to the tolerant variety. Increase in ascorbate-glutathione cycle enzymes activities, CAT, ascorbate-glutathione content, SOD, and ascorbate-glutathione redox clearly evident that CAT, ascorbate-glutathione cycle and SOD played a significant activity in ROS detoxification of tolerant *A. tricolor* variety.

## Introduction

Drought stress causes oxidative stress by decreasing stomatal conductivity that confines CO_2_ influx in to the leaves. This reduces the leaf internal CO_2_, which leads to the formation of ROS such as hydroxyl radicals (OH•) singlet oxygen (^1^O_2_), hydrogen peroxide (H_2_O_2_), alkoxyl radical (RO) and superoxide radical (O_2_^•−^) mainly by enhancing electrons leakage to oxygen molecule^1–4^. In plant cell, mitochondria, chloroplasts and peroxisomes are the main locations of ROS generation^5^. In addition, Environmental stress stimulates xanthine oxidase in peroxisomes, amine oxidase in the apoplast and NADPH oxidases (NOX) in the plasma membrane and produce ROS^6,7^. Environmental stress induces excess ROS that can injure plant cells by oxidation of cellular components such as proteins, inactivate metabolic enzymes, DNA and lipids^8,9^.

The response of plant defense system to stress varies with the times, duration of contact and stress severity, type of organ or tissue and developmental stage^10,11^. At a certain level, ROS works as an indicator molecule for activating acclimatory/protection responses through transduction pathways, where H_2_O_2_ acts as a secondary messenger^12,13^. However, additional ROS induces harmful effects on plant cells. As a result, defenses against ROS are activated^14^ by an array of nonenzymatic antioxidants [metabolites such as ascorbate (AsA), carotenoids, glutathione (GSH) and proline] and antioxidant enzymes [such as guaiacol peroxidases (GPOX), catalase (CAT), superoxide dismutase (SOD) and AsA-GSH cycle enzymes like glutathione reductase (GR) ascorbate peroxidase (APX), monodehydroascorbate reductase (MDHAR), dehydroascorbate reductase (DHAR)], work together for detoxification of ROS^7,8,15–20^. In glutathione-ascorbate cycle, reduced glutathione is produced from oxidized glutathione through the donated electrons of all nonenzymatic and enzymatic antioxidants^8^. In addition to their damaging effects on cells, ROS can also take part as signaling molecules in many biological processes such as growth, enclosure of stomata, stress signaling and development^9,21–23^. Recently more attention has been given to understand the antioxidant defense mechanism in plants exposed to drought stress^24–26^. Abiotic stress enhances the production of AsA–GSH and AsA–GSH cycle enzymes activities for cellular protection. Plant water relations play a significant role in the stimulation and/or modulation of antioxidative defense mechanism at drought stress^27–29^.

In Bangladesh, *A. tricolor* is very cheap and common leafy vegetable. It grows widely in Southeast Asia, Africa, arid and semiarid regions around the globe. There is no information on mechanism of water deficit tolerance of *A. tricolor* genotypes in relations to antioxidative defense system in ROS detoxification. In our previous studies^30–37^ we selected some high yielding potential genotypes rich in antioxidant content. We also found tremendous increment of ascorbic acid under drought^38^ and salinity^39^ stress and APX^40^ with the severity of drought stress in selected genotypes. This result grew many interests to study the role of antioxidant enzymes especially AsA-GSH cycle pathway for enhancing the protection of *A. tricolor* from oxidative stress under drought stress. In this study, we want to elucidate key physiological, enzymatic and non-enzymatic pathways involved in ROS detoxification and tolerance of *A. tricolor* under drought stress.

## Results

Variety, drought stress, and variety × drought stress interactions were significantly different for all the studied traits (*P* > 0.01).

### Response to drought stresses on plant growth, photosynthetic pigment, and relative water content

**Figure 1:**
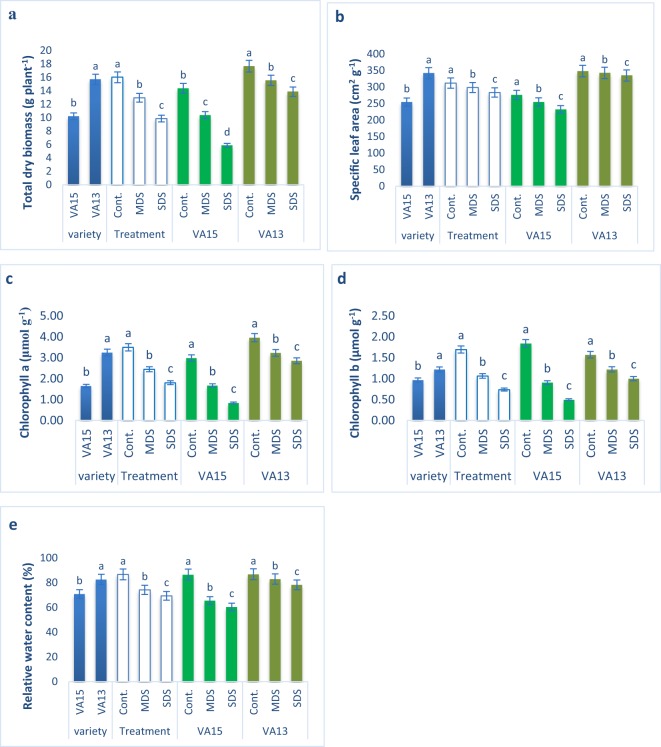
Effect of drought stress on growth, photosynthetic pigment biosynthesis and leaf relative water content (RWC%) in *A. tricolor*. Cont., control (100% FC); MDS (60% FC), moderate drought stress; SDS (30% FC), severe drought stress; total dry biomass (**a**); specific leaf area (**b**); chlorophyll *a* (**c**); chlorophyll *b* (**d**) and leaf relative water content (**e**); Values are mean ± SD of four replicates and different letters are differed significantly by Duncan Multiple Range Test (P < 0.01).

The major growth parameters, such as total biomass and specific leaf area (SLA); photosynthetic pigment biosynthesis, such as leaf relative water content, chlorophyll *a* and chlorophyll *b* of both varieties reduced significantly under moderate drought stress (MDS) and severe drought stress (SDS) conditions compared to control condition (Fig. 1a–e). The decline in total biomass, specific leaf areas, chlorophyll *b*, chlorophyll *a* content and RWC of VA15 were much greater compared to VA13 in all the treatments (Fig. 1a–e). Total biomass, specific leaf area, chlorophyll *a*, chlorophyll *b* content and RWC of VA15 were declined by 28%, 8%, 44%, 71% and 24% under MDS and 59%, 16%, 58%, 56% and 30% under SDS conditions, while total biomass, specific leaf area, chlorophyll *a*, chlorophyll *b* content and RWC of VA13 were declined by 12%, 2%, 18%, 28% and 5% under MDS and 21%, 4%, 8%, 19% and 10% under SDS conditions, respectively compared to control conditions.

### Influence of drought stresses on lipid peroxidation, hydrogen peroxide, and EL%

**Figure 2:**
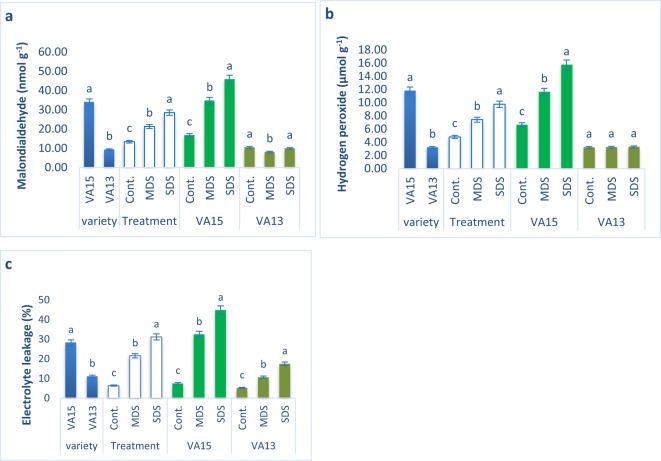
Influence of drought stress on malondialdehyde content (MDA, **a**); hydrogen peroxide (H_2_O_2_, **b**), electrolyte leakage (EL%, **c**) in *A. tricolor*. Cont., control (100% FC); MDS (60% FC), moderate drought stress; SDS (30% FC), severe drought stress; Values are mean ± SD of four replicates and different letters are differed significantly by Duncan Multiple Range Test (P < 0.01).

MDA, H_2_O_2_ content and EL% augmented progressively with the increment of drought stress in the sensitive variety VA15 under MDS and SDS conditions, whereas the increments of EL% in the tolerant variety VA13 under MDS and SDS conditions were much lower compared to control condition. In contrast, there were no increments of MDA and H_2_O_2_ content in the tolerant variety VA13 under MDS and SDS conditions compared to control treatment. (Fig. 2a–c). EL% in the tolerant variety VA13 were increased by 103% under MDS and 233% under SDS conditions, while MDA, H_2_O_2_ content and EL% of sensitive variety VA15 were rapidly increased by 107%, 76%, and 331% under MDS and 173%, 137% and 495% under SDS conditions, compared to control conditions, respectively.

### Effect of drought stresses on proline, total carotenoids, ascorbate, glutathione content

**Figure 3:**
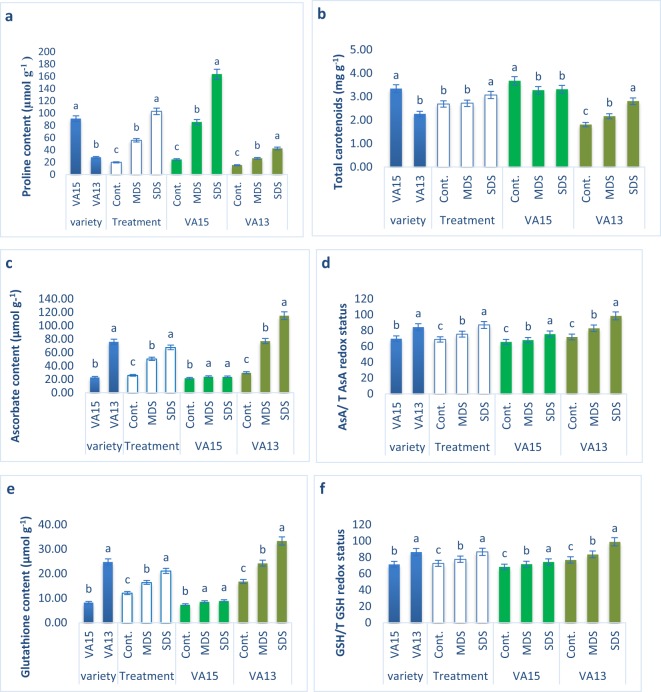
Effect of drought stress on proline content (**a**); total carotenoid (**b**); ascorbate content (**c**); ascorbate/total ascorbate% (**d**); glutathione content (GSH) (**e**); glutathione/total glutathione% (**f**) in *A. tricolor*. Cont., control (100% FC); MDS (60% FC), moderate drought stress; SDS (30% FC), severe drought stress; Values are mean ± SD of four replicates and different letters are differed significantly by Duncan Multiple Range Test (P < 0.01).

Proline content was augmented significantly with the increment of drought stress in VA15 under MDS and SDS conditions, while total carotenoids reduced from control to MDS and which was statistically similar at MDS and SDS conditions. Proline increments in VA13 under MDS and SDS conditions were comparatively much lower than in VA15 compared to control condition, while total carotenoids increment in VA13 under MDS and SDS conditions were comparatively higher than in VA15 compared to control condition (Fig. 3a,b). Proline of VA15 was increased by 248% under MDS and 566% under SDS conditions, respectively when compared with control treatment. In contrast, proline and total carotenoids of VA13 were increased by 72% and 20% under MDS and 176% and 55% under SDS conditions, respectively in comparison with control treatment. Ascorbate, ascorbate/total ascorbate redox status, glutathione and glutathione/total glutathione redox status remarkably augmented with the increment of drought stress in VA13 under MDS and SDS conditions, while ascorbate, ascorbate redox, glutathione and glutathione redox increments in VA15 under MDS and SDS conditions were much lower than in VA13 compared to control condition, respectively (Fig. 3c–f). Ascorbate, ascorbate redox, glutathione and glutathione redox of VA13 were increased by 158% 15%, 45% and 9% under MDS and 286% 37% 98% and 29% under SDS conditions, whereas ascorbate, ascorbate redox, glutathione and glutathione redox of VA15 were increased by 11% 19% 16% and 5% under MDS and 10% 30% 21% and 9% under SDS conditions, respectively compared to control conditions.

### Effect of drought stresses on antioxidant enzymes activities

**Figure 4:**
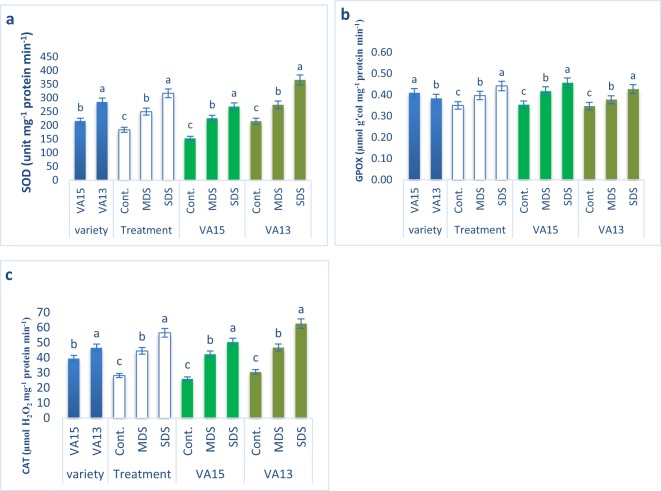
Response of (**a**), superoxide dismutase (SOD) (unit mg^−1^ protein min^−1^); (**b**), guaiacol peroxidase (GPOX) (µmol g’col mg^−1^ protein min^−1^); (**c**), catalase (CAT) (µmol H_2_O_2_ mg^−1^ protein min^−1^) enzymes to drought stress in *A. tricolor*. Cont., control (100% FC); MDS (60% FC), moderate drought stress; SDS (30% FC), severe drought stress; Values are mean ± SD of four replicates and different letters are differed significantly by Duncan Multiple Range Test (P < 0.01).

CAT and SOD activities progressively augmented with the increment of drought stress under MDS and SDS conditions in comparison with control treatment in both varieties, however, the increments of SOD and CAT activities in VA13 were higher compared to VA15 at all drought stress levels (Fig. 4a,c). CAT and SOD activities of VA13 were increased by 28% and 53% under MDS and 70% and 105% under SDS conditions, whereas CAT and SOD activities of VA15 were increased by 48% and 64% under MDS and 76% and 94% under SDS conditions, respectively compared to control treatment. The GPOX activity significantly and remarkably augmented with the increment of drought stress under MDS and SDS conditions in comparison with control treatment in both varieties, while VA15 exhibited the highest increments compared to VA13 at all drought stress treatment (Fig. 4b). The GPOX activity of VA13 was increased by 9% and 23% at MDS and SDS conditions, whereas GPOX activity of VA15 was increased by 18% and 29% at MDS and SDS conditions, respectively in comparison with control treatment.

### Effect of drought stresses on AsA-GSH cycle enzymes activities

**Figure 5:**
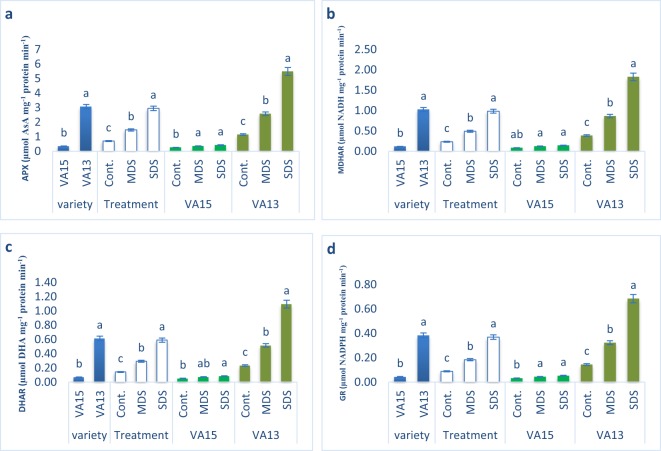
Response of ascorbate-glutathione cycle enzymes to drought stress in *A. tricolor*. Cont., control (100% FC); MDS (60% FC), moderate drought stress; SDS (30% FC), severe drought stress; [9a, ascorbate peroxidase (APX) (µmol AsA mg^−1^ protein min^−1^); (**b**), monodehydroascorbate reductase (MDHAR) (µmol NADH mg^−1^ protein min^−1^); (**c**), dehydroascorbate reductase (DHAR) (µmol DHA mg^−1^ protein min^−1^); (**d**), glutathione reductase (GR) (µmol NADPH mg^−1^ protein min^−1^]; Values are mean ± SD of four replicates and different letters are differed significantly by Duncan Multiple Range Test (P < 0.01).

MDHAR, DHAR, APX and GR activity progressively augmented with the increment of drought stress under MDS and SDS conditions in comparison with control treatment in the tolerant genotype VA13, while the increments of those enzymes’ activities were much lower in the sensitive genotype VA15 compared to tolerant genotype VA13 at all drought stress levels (Fig. 5a–d). MDHAR, DHAR, APX and GR activity of VA13 were augmented by 125% 125%, 122% and 124% under MDS and 379%, 375%, 371% and 375% under SDS conditions, whereas MDHAR, DHAR, APX and GR activity of VA15 were augmented by 45% 40%, 37% and 2% under MDS and 70%, 63%, 64% & 20% under SDS conditions, respectively compared to control condition.

## Discussion

The results of the present investigation suggested that *A. tricolor* is tolerant to drought stress. We select one tolerant and one sensitive *A. tricolor* genotype previously screened for drought stress based on morphological and physiological traits to elucidate key non-enzymatic, physiological, and antioxidant enzymatic defense mechanisms involved. The above defense mechanisms significantly varied in the tolerant and sensitive varieties as are discussed in detail in the following sections.

### Mechanisms of differential adaptation to drought stress

Growth is a primary process that affects drought^4^. Total biomass of both varieties of *A. tricolor* significantly declined to MDS and SDS conditions, in comparison with control treatment, indicating that drought stress declined the growth of both varieties. Whereas the tolerant variety showed less decline in total biomass. These results were in full agreement with the results of Sekmen *et al*.^41^ who observed that the growth rate of tolerant M-503 cultivar was less affected from drought treatments as compared to the sensitive 84-S cultivar. In our earlier study, we observed decrease in RWC and biomass reduction with the increment of drought stress^40^. Previous studies also have shown that drought stress inhibited growth and RWC in strawberry^16^, xerophyte *Capparis ovata*^42^ and cotton^41^. It might be accredited to prevent cell elongation and expansion^43,44^, reduction of turgor pressure, changes of energy from growth to biosynthesis of metabolites to preserve turgor pressure of cell, declines in absorption of water that ultimately reduces water content of cell and nitrogen assimilation^45,46^, reduces the photo-assimilation^47^ and metabolites for cell division^48^. In the present investigation, an indicator of leaf thickness SLA had a sharp decline with the increment of drought stress in both varieties under MDS and SDS conditions in comparison with control treatment. Guerfel *et al*. in olive and Sarker and Oba in *Amaranthus*^40^ observed a similar trend of decline in SLA^49^. Growth and SLA reduction in sensitive genotype VA15 were significantly higher than that of tolerant genotype VA13 under both MDS and SDS conditions i.e., VA13 showed better adaptation compared to VA15. Similarly, Zheng *et al*.^50^ also found different adaptation in two genotypes of *C. bungee*. In this study, VA13 had more chlorophylls content and less decline in chlorophylls than VA15, suggesting that VA13 was more drought tolerant compared to VA15. Sarker and Oba^40^ in *A. tricolor*, Shahbaz *et al*.^51^ in wheat and Zhang and Kirkham^52^ in sorghum and sunflower observed a decline in leaf chlorophyll contents under drought stress conditions. Drought stress induces the oxidation of chlorophyll pigment resulting in decrement of chlorophyll pigments^53^, chloroplasts disruption or augmented activity of chlorophyllase^54^. *A. tricolor* has betacyanin and betaxanthin that absorb a substantial amount of radiation which ultimately protects chloroplasts from harmful excessive light under stressful condition^55^. In the present study, this might be the reason for lower chlorophyll reduction in both varieties. RWC is convenient attributes for assessing physiological hydration condition of crops and its metabolism and existence. It might be utilized for distinguishing between sensitivity and tolerance in drought-stressed crops^56^. Both *A. tricolor* varieties resulted in a drought-induced reduction of RWC under MDS and SDS conditions, compared to the control treatment, respectively, however, the reduction was more drastic in VA15 compared to VA13. In our previous studies, we also found similar results in *A. tricolor*^40^. Munne-Bosch and Penuelas^16^ in strawberry, Ozkur *et al*.^42^ in xerophyte *Capparis ovata* and Sekmen *et al*.^41^ in cotton observed a similar decline in RWC under drought stress. Drought stress reduces turgor pressure, decreases available water in the soil, hampers roots water absorption, finally results in decrease in RWC of leaves. Under drought stress, VA13 exhibited higher RWC; it might be due to probable osmoregulation approach and greater antioxidants accumulation under drought stress in comparison to VA15^40^. Turkan *et al*.^57^ and Cia *et al*.^58^ showed that tolerant varieties have maintained better RWC under drought stress. Thus, VA13 seemed to be more capable to decrease the cellular osmotic pressure and to permit the roots for absorbing adequate water to sustain cell turgor pressure and for taming potentiality against hydration status.

### Differential ROS association with drought sensitivity and tolerance in *A. tricolor*

Drought stresses intensify the manufacture of ROS like alkoxy radicals, O_2_^•−^, singlet oxygen, H_2_O_2_, OH• etc. which ultimately create oxidative stress in cell^40,59^. Primary stimulation of O_2_ by xanthine oxidase, O_2_^•−^ dismutation, electron reduction at higher O_2_ level are the main mechanisms of ROS generation in plants^60^. ROS causes oxidative stress through damage DNA, lipids and proteins, restricting the normal cell functions. Drought stress aggravates ROS production in chloroplasts, mitochondria and peroxisomes^5,48^. In our study, we found a substantial production of H_2_O_2_, lipid peroxidation and increase in EL in the sensitive variety (VA15) of *A. tricolor* under drought stress. EL leakage was much greater in the sensitive variety (VA15) as compared to the tolerant variety (VA13). These results agreed with the results of our previous study in amaranth^40^, Christou *et al*.^61^ in strawberry and Chakraborty *et al*.^60^ in groundnut. Our results clearly demonstrated that at similar drought stress, the sensitive *A. tricolor* accumulated more ROS compared to the tolerant variety. Hence the tolerant variety maintained the ROS to a relatively lower level than sensitive variety. In the present investigation, extreme accumulation of H_2_O_2_ at MDS and SDS in the sensitive variety might be due to acceleration of the Haber-Weiss reaction that causing formation of hydroxyl radical (•OH), hence, resulting in more MDA production and damage of cell membrane^8^. At stressful conditions, it is crucial to maintaining a balance between ROS assembly and detoxification^62^. In our study, drought-stressed conditions remarkably augmented non-enzymatic and enzymatic antioxidants by defensive techniques from MDS to SDS to lessen EL, H_2_O_2_ and MDA accumulation. The tolerant cultivars VA13 had very low H_2_O_2_ and MDA content. The tolerant cultivar improved the stressful condition by several protection ways, such as non-enzymatic antioxidant, antioxidant enzymes and AsA-GSH cycle which inhibited drought stress impact by protection of ROS generation. Under water stress, electrolyte leakage is considered to be a symbol of damage and descent^63^. In the present investigation, drought stress progressively enhanced electrolyte leakage. Hence, electrolyte leakage might be used to distinguish stress-susceptible and tolerant cultivars. Abiotic stress tolerance is associated with lower electrolyte leakage. The Severity of drought-induced progressive increment in MDA and H_2_O_2_ that enhanced the damage of cell membrane in the sensitive variety and demonstrated by a sharp increase in EL. Tolerant genotype VA13 showed lower electrolyte leakage compared to sensitive genotype.

### Role of proline and antioxidants to supplement enzymatic ROS detoxification

Proline content of both the varieties was significantly increased under MDS and SDS conditions, whereas the increment was greater in the sensitive variety VA15 compared to tolerant variety VA13. It is evident from the results that proline had no significant role in the mechanisms of drought stress tolerance in *A. tricolor*, as a functional osmolyte and antioxidant for adjustment of osmotic stress and ROS detoxification in *A. tricolor* as it accumulates to higher levels in the drought-sensitive variety. Nayyar and Walia^64^ and Tatar and Gevrek^65^ in wheat, Zheng *et al*.^50^ *Catalpa bungee* observed proline increment under drought stress. Carotenoids are capable to scavenge lipid peroxy-radicals and singlet oxygen and inhibit superoxide generation and lipid peroxidation under drought stress^48^. Total carotenoids are lipophilic antioxidants that are capable to purify different types of ROS^66^. In plants, total carotenoid usually absorbs light at 400 and 550 nm and transfer the apprehended energy to the chlorophyll^67^. Carotenoids can act as an antioxidant that inhibits oxidative damage by scavenging ^1^O_2_, quenching triplet sensitizer (3Chl*), exciting chlorophyll (Chl*) and protecting the photosynthetic apparatus. Ascorbate (AsA) is one of the powerful antioxidants^68^. AsA and αtocopherols predominately quench O_2_ straightly or by enzymes catalysis. It permits non-enzymatic and enzymatic antioxidative ROS detoxification. AsA scavenges OH, SOR and ^1^O_2_ directly and reduces H_2_O_2_ to water through ascorbate peroxidase reaction^69^. Antioxidant ascorbate and total carotenoid had a vital role in counterbalancing oxidative stress and manipulating homeostasis of ROS in plants^70^. Our results showed that the total carotenoid level was increased in VA13, while the decrement of this compound was observed in VA15. In the tolerant variety VA13, had a remarkable rise in ascorbate-glutathione content and ascorbate-glutathione redox status, while the sensitive variety VA15 exhibited negligible increment of ascorbate-glutathione content and ascorbate-glutathione redox status. For instance, drought and salt stress increased the activity of ascorbate-glutathione content and ascorbate-glutathione redox status in pea^71^, wheat^72^, sorghum and sunflower^52^, *Catalpa bungee*^50^, strawberry^16^ and groundnut^60^, particularly for tolerant lines under water deprivation condition. The AsA-GSH content, AsA-GSH redox status specifies the essential part of the AsA–GSH cycle for detoxification of ROS in the tolerant *A. tricolor*. Similarly, Hernandez *et al*.^71^ reported that salinity stress accumulated higher transcripts of the AsA–GSH cycle in the tolerant variety compared to the sensitive variety.

### SOD, CAT and AsA–GSH cycle enzymes predominately confer drought tolerance in *A. tricolor*

Drought stress generated superoxide from photosynthetic and respiratory electron leakage in chloroplast. Superoxide dismutase (SOD) enzyme dismutated superoxide into H_2_O_2_. H_2_O_2_ was decomposed by different peroxidases such as ascorbate peroxidase (APX), glutathione peroxidase (GPX) and phenol peroxidase^5^ into the water by using various reducing agents. In contrast, catalase (CAT) mostly decomposed photorespiration mediated H_2_O_2_ in the peroxisome^10^.

In this study, we found that drought stress induced CAT and SOD activities in both varieties whereas, CAT and SOD activities were much greater in the tolerant variety VA13 compared to sensitive variety VA15, suggesting role of CAT and SOD in drought tolerance in *A tricolor* by detoxification of H_2_O_2_ and activating dismutation reaction to alter SOR to hydrogen peroxide_,_ respectively. These results agreed to results of Ben Amor *et al*. in halophyte *Cakile maritima*^73^ where they interrelated in increased SOD activity with plant salt tolerance. Khanna-Chopra and selote^72^ in wheat, Ozkur *et al*.^42^ in *Capparis ovata*, Zhang and Kirkham^52^ in sorghum and sunflower and Chakraborty *et al*.^60^ in groundnut observed enhanced activities of SOD, POX and CAT under drought and salt stress. Sekmen *et al*.^41^ found that the sensitive genotype 84-S associated with decreased activities of catalase (CAT) and peroxidase (POX) to combined stress while the tolerant genotype M-503 was associated with higher activities of superoxide dismutase (SOD) and ascorbate peroxidase (APX) and induced CAT and POX at combined drought and heat stress. In contrast, GPOX had significant and remarkable increasing activity under drought stress, in both varieties, while sensitive variety, VA15 exhibited the highest increase compared to VA13 at all drought stress treatments. Drought stress accelerated higher GPOX increase in the sensitive variety compared to the tolerant variety; it is clearly evident that GPOX had a significant role in enhancing APX activity in the sensitive variety at greater H_2_O_2_ concentration.

There was a slight and negligible increase in GR, MDHAR, APX and DHAR activity in sensitive variety VA15 under drought stress, while tolerant variety VA13 exhibited the greatest dramatic increase in GR, MDHAR, APX and DHAR activity under drought stress. Hernandez *et al*.^71^ in pea found increased activities of GR, MDHAR, APX and DHAR while Chakraborty *et al*.^60^ in groundnut showed APX increment under salt stress. Similarly, Khanna-Chopra and selote^72^ and Ozkur *et al*.^42^ found that increased activities of APX and GR were associated with drought stress. It indicated that at lower H_2_O_2_ load, GR, MDHAR, APX and DHAR performed as a main ROS scavenging enzyme in *A. tricolor* under drought stress that may have related to satisfactory regulation of H_2_O_2_ in the tolerant variety VA13. Increase in AsA-GSH content, reduced AsA-GSH redox status accompanied by AsA–GSH cycle enzymes such as GR, MDHAR, APX and DHAR, clearly evident that AsA–GSH cycle played a crucial role for scavenging ROS in the tolerant variety of *A. tricolor*. Abogadallah *et al*.^74^ reported APX-GR as the main H_2_O_2_ detoxifier at low H_2_O_2_ load and performed as a satisfactory controller for ROS balancing in barnyard grass under salt stress.

The present study concluded that drought stress exhibited differential responses to tolerant and sensitive *A. tricolor* genotypes in terms of growth, physiological, enzymatic and non-enzymatic ROS detoxification pathways involved in the tolerance of *A. tricolor*. Better growth, photosynthetic pigments, RWC, and lower ROS concentration and EL in the tolerant genotype can be recognized to better antioxidative enzymatic protection and cellular antioxidant pool, such as AsA-GSH content, AsA-GSH redox. The present investigation revealed that *A. tricolor* genotype doesn’t certainly require concurrent initiation of all antioxidant enzymes for drought tolerance. Only SOD, CAT and AsA-GSH cycle enzymes play a vital role in major ROS detoxification in the tolerant amaranth genotype. Increase in CAT, AsA-GSH content, SOD, AsA-GSH redox and AsA-GSH cycle enzymes activities, clearly evident that AsA–GSH cycle, SOD and CAT play a crucial role in tolerance of *A. tricolor*.

## Methods

### Plant materials and experimental conditions

We selected one drought tolerant (VA13) and one moderately drought sensitive (VA15) *Amaranthus tricolor* varieties on the basis of our previous morphological and physiological study (Data not published). These two varieties were grown in pots of a rain shelter open field of Bangabandhu Sheikh Mujibur Rahman Agricultural University, Bangladesh (AEZ-28, 24°23′ north latitude, 90°08′ east longitudes, 8.4 m.s.l.). The trial area remains covered during rainfall events and otherwise exposes plants to ambient field conditions. Topsoil layer of the experimental station was collected from 30 cm depths for the potting soil. The soil was silty clay with slightly acidic (pH 6.4) and low in organic matter (0.87%), total N (0.09%) and exchangeable K (0.13 c mol kg^−1^). The soil S content was at par with critical level, while P and Zn contents were above the critical level (Critical levels of P, S, and Zn were 14, 14 and 0.2 mg kg^−1^, respectively and that of K was 0.2 c mol kg^−1^). The seeds were sown in plastic pots (22 cm in height and 60 cm length and 40 cm width) maintaining 20 cm apart rows and 5 cm from plant to plant distance. The experiment comprised two factors (drought level and genotype) in a factorial fashion in a randomized complete block design (RCBD) with four replications. Total 36 pots were sown with 18 pots per variety and 12 pots per treatment. Fertilizer was applied to the rate of 92:48:60 kg ha^−1^ N:P_2_O_5_:K_2_O as a split dose. First, in pot soil, at the rate of 46:48:60 kg ha^−1^ N:P_2_O_5_:K_2_O and second, at 10 days after sowing (DAS) at the rate of 46:0:0 kg ha^−1^ N:P_2_O_5_:K_2_O. The average day/night temperatures, relative humidity and day length during the experimental period was 25/21 °C, 74%, and 12 h, respectively. Each variety was grouped into four sets and subjected to three drought stress treatments that are, control (Cont., 100% FC); moderate drought stress (MDS, 60% FC); and severe drought stress (SDS, 30% FC). At first, pot soil field capacity was measured by the gravimetric method. Then the amount of water at field capacity was measured by subtracting the weight of completely dry soil from the weight of soil at field capacity. Pot weight (including pot soil) for each treatment was calculated by weighing of completely dry soil and amount of water required for attaining respective field capacity. Pots were well-irrigated every day up to 10 DAS for dynamic growth and proper establishment of seedlings. Imposition of water stress treatment was started at 25 DAS. Pots were weighed twice a day at 12 h intervals. To achieve the target field capacity of each water condition, the amount of water equaling that lost through transpiration and soil evaporation, percolation and leaching were added. Water stress was imposed up to 55 DAS. The leaves of *A. tricolor* were harvested at 55 DAS. Sampling was completed between 11:00 and 12:00. For quantification of plant parameters, fully emerged top young leaves from control and stressed plants were sampled. All the parameters were measured in four replicates.

### Plant growth measurements

At 55 DAS, total biomass and SLA were measured from 5 plants. LI-3100 leaf area meter (LICOR. Inc., Lincoln, NE, USA) was used to determine total leaf area per plant. the samples were oven dried at 70 °C until constant weight achieved. The dry mass of total plant and leaves was taken. For determination of SLA, total plant leaf area was divided by the leaf dry weight.

### Determination of chlorophylls and total carotenoid content

80% acetone extracts were used to determine chlorophyll *a*, chlorophyll *b* and total carotenoid from fresh amaranth leaves according to the method of Lichtenthaler and Wellburn method^75^. The absorbance was taken at 663, 646 and 470 nm, respectively using spectrophotometer (Hitachi, U-1800, Tokyo, Japan). Data were calculated as μmoles chlorophyll per g and mg total carotenoid per g dry weight (dw), respectively.

### Determination of leaf relative water content

Barrs and Weatherley’s method was followed to determine leaf relative water content (RWC)^76^. RWC was determined from fully expanded leaves of three plants per replicate. From the interveinal area of each plant, three leaf discs (10 mm in diameter) were punched using a cork borer. The fresh mass (FW) of pooled discs per replicate were determined immediately. To avoid respiratory losses, weighed leaf discs were then placed in distilled water for 4 hours at 20 °C under dim illumination. The leaf discs were floated for four hours for complete hydration. The leaf discs were then carefully blotted to remove surface water. Turgid mass (TW) were taken to calculate water uptake. Dry mass (DW) of the leaf discs was determined by drying the tissues at 70 °C for 2 to 4 d. RWC was measured as (FW − DW)/(TW − DW) × 100.

### Determination of leaf malondialdehyde and H_2_O_2_

2-thiobarbituric acid (TBA) was used to determine malondialdehyde (MDA) following the method of Hodges *et al*.^77^. Briefly, 5 ml 0.6% TBA in 10% trichloroacetic acid (TCA) was added in 1 g mortar and pestle grounded fresh vegetable amaranth leaf. The mixture was heated at 100 °C for 15 min and then cooled in ice. Finally, the mixture was centrifuged at 5000 rpm/min for 10 min. The absorbance was taken at 450, 532, and 600 nm. The MDA content was determined on a fresh weight basis as follows:

$${\rm{MDA}}({\rm{\mu }}\mathrm{mol}\,{{\rm{g}}}^{-1}{\rm{FW}})=6.45({\rm{OD}}532-{\rm{OD}}600)-0.56{\rm{OD}}450$$

Data were calculated as nmoles per gram dry weight (nmol g^−1^ dw).

KI was used to determine hydrogen peroxide^78^. 0.5 ml 0.1%, trichloroacetic acid (TCA), leaf extract supernatant, 0.5 ml of 100 mM potassium phosphate buffer, and 2 ml reagent (1 ml KI w/v double-distilled water) were added in the reaction mixture. 0.1% TCA was added instead of leaf extract for blank probe. The reaction was kept in the dark for 1 h, and absorbance was taken at 390 nm. A standard curve was used to determine the amount of hydrogen peroxides from known H_2_O_2_ concentrations. Data were calculated as μmoles per gram dry weight (μmol g^−1^ dw).

### Determination of electrolyte leakage

Electrolyte leakage (EL) was measured following the method of Lutts *et al*.^79^. Six randomly chosen plants per treatment (four mature leaves per plant) were taken and cut into 1 cm segments. Leaf samples were washed three times with distilled water to remove surface contamination and then placed in individual stopper vials containing 10 mL of distilled water. The samples were incubated at room temperature (25 °C) on a shaker (100 rpm) for 24 h. The electrical conductivity of the bathing solution (EC1) was read after incubation. The same samples were then placed in an autoclave at 120 °C for 20 min and a second reading of the EC (EC2) was made after cooling the solution to room temperature. The EL was calculated as EC1/EC2 and expressed as the percentage.

### Proline and antioxidant

3% sulfosalicylic and ninhydrin extraction buffers were used to measure proline content from freeze-dried leaves^80^. 0.04 g dry leaves were homogenized with 3% (w/v) sulfosalicylic acid and centrifuged at 3000 × g for 10 min. 400 μl of the reagent mixture (30 ml glacial acetic acid, 20 ml phosphoric acid and 1.25 g ninhydrin) was mixed with 200 μl supernatant and heated in sealed test tubes at 100 °C for 1 h. 4 ml toluene was added to each cooled sample. The absorbance was measured using a Hitachi, U-1800, Tokyo, Japan spectrophotometer at 520 nm. Data were calculated as μmoles per gram dry weight (μmol g^−1^ dw).

Leaf samples were prepared for AsA, DHA, GSH and GSSG analyses by homogenizing 1 g leaf material (F. wt.) in 10 ml of cold 5% sulphosalicylic acid^52^. The homogenate was centrifuged at 22000 × g for 15 min at 4 °C, and the supernatant was collected for analyses of ascorbate and glutathione. AsA, DHA and total ascorbate (AsA + DAsA) were measured according to Zhang and Kirkham^52^. DHA was reduced to AsA by adding DTT and total ascorbate was measured. The concentration of DHA was estimated from the difference between total ascorbate and AsA. 0.3 ml aliquots of the supernatant, 0.75 ml of 150 mM phosphate buffer (pH 7.4) containing 5 mM EDTA, and 0.15 ml of 10 mM DTT were added to determine total ascorbate. To remove excess DTT, 0.15 ml of 0.5% *N*-ethylmaleimide was added after incubation for 10 min at room temperature. Instead of DTT and N-ethylmaleimide 0.3 ml H_2_O was added to measure in a similar reaction mixture. After adding 0.6 ml of 10% TCA, 0.6 ml of 44%, orthophosphoric acid, 0.6 ml 4% *α*,*α*′-dipyridyl 70% ethanol, and 0.3% (w/v) FeCl3 reagents color was developed in both reaction mixtures. After vortex mixing, the mixture was incubated at 40 °C for 40 min and the *A*_525_ was read. A standard curve in the range 0–100 µg AsA ml^−1^ was prepared. Data were calculated as μmoles per gram dry weight (μmol g^−1^ dw).

GSH and GSSG were assayed according to the methods of Zhang and Kirkham^52^. One ml aliquot of the supernatant was neutralized with 1.5 ml of 0.5 M phosphate buffers (pH 7.5), then 50 µl H_2_O was added; this sample was used for the assay of total glutathione (GSH + GSSG). Another 1 ml aliquot of the supernatant was neutralized with 1.5 ml of 0.5 M phosphate buffers, 50 µl of 2-vinylpyridine was added to mask GSH, and the constants of the tube were mixed until an emulsion formed. The tube was then incubated for 60 min at room temperature. This sample was used for the assay of GSSG. GSH was estimated as the difference between total glutathione and GSSG. Glutathione content was measured in a 3 ml reaction mixture containing 0.2 mM NADPH, 100 mM phosphate buffer (pH 7.5), 5 mM EDTA. 0.6 mM DTNB and 3 units of GR. The reaction was started by adding 0.l ml of extract sample obtained as described above. The reaction rate was monitored by measuring the change in absorbance at 412 nm for 1 min. A standard curve was developed based on GSH in the range 0–50 µmol ml^−1^. Data were calculated as μmoles per gram dry weight (μmol g^−1^ dw).

### Determination of soluble protein content

dye-binding method and bovine serum albumin as standard were used to determine soluble proteins. Absorbance was read through a spectrophotometer at 595 nm^81^.

### Determination of antioxidant enzymes activities

1 g of leaf samples were freezed in liquid nitrogen followed by grinding in 10 mL extraction buffer (0.1 M phosphate buffer, pH 7.5, containing 0.5 mM EDTA in case of SOD, GPOX, CAT and 1 mM ascorbic acid in case of APX to prepare the extract. The homogenates were filtered through four layers of cheesecloth and then centrifuged at 4 °C for 20 min at 15000 × g. The supernatant was collected and used for the assays of enzymatic activities. All steps in the preparation of the enzyme extract were carried out at 4 °C.

Total SOD (EC 1.15.1.1) activity was estimated by the inhibition of the photochemical reduction of nitroblue tetrazolium (NBT) by the enzyme^82^. 2 mM riboflavin (0.1 mL) was added in 3 mL of reaction mixture (13.33 mM methionine, 75 µM NBT, 0.1 mM EDTA, 50 mM phosphate buffer (pH 7.8), 50 mM sodium carbonate, 0.1 mL enzyme extract) and placing the tubes under two 15 W fluorescent lamps for 15 min to start the reaction. The absorbance was recorded at 560 nm, and one unit of enzyme activity was taken as that amount of enzyme, which reduced the absorbance reading to 50% in comparison with tubes lacking enzyme.

Guaiacol peroxidase GPOX (EC 1.11.1.7) activity was measured in terms of increase in absorbance due to the formation of tetra-guaiacol at 470 nm and the enzyme activity was calculated as per extinction coefficient of its oxidation product, tetra-guaiacol ε = 26.6 mM^−1^ cm^−1^ ^83^. 50 mM phosphate buffer (pH 6.1), 16 mM guaiacol, 2 mM H_2_O_2_ and 0.1 mL enzyme extract were mixed in the reaction mixture. The mixture was diluted with distilled water to make up the final volume of 3.0 mL. Enzyme specific activity is expressed as µmol tetra-guaiacol formed per min per mg protein.

Catalase (EC 1.11.1.6) was assayed by measuring the disappearance of H_2_O_2_^84^. 0.5 mL of 75 mM H_2_O_2_ was added in 1.5 mL of 0.1 M phosphate buffer (pH 7) and 50 µL of diluted enzyme extract in 3 mL reaction mixture. The decrease in absorbance at 240 nm was observed for 1 min and enzyme activity was computed by calculating the amount of H_2_O_2_ decomposed.

Ascorbate peroxidase (EC 1.11.1.1) was assayed by recording the decrease in optical density due to ascorbic acid at 290 nm^85^. 50 mM potassium phosphate buffer (pH 7.0), 0.5 mM ascorbic acid, 0.1 mM EDTA, 0.1 mM H_2_O_2_, 0.1 mL enzyme and water to make a final volume of 3.0 mL in which 0.1 mL of H_2_O_2_ was added to initiate the reaction. The decrease in absorbance was measured spectrophotometrically and the activity was expressed by calculating the decrease in ascorbic acid content using a standard curve drawn with identified concentrations of ascorbic acid.

1 ml of 50 mM potassium phosphate buffer (pH 7.0), containing 10% (w/v) polyvinylpyrrolidone (PVP), 0.25% (v/v) Triton X-100, 1 mM phenylmethylsulfonyl fluoride (PMSF) and 1 mM ASA were added in a homogenate of 100 mg (FW) of leaf tissues to measure glutathione reductase (GR, EC 1.6.4.2), dehydroascorbate reductase (DHAR, EC 1.8.5.1), and monodehydroascorbate reductase (MDHAR, EC 1.6.5.4). Murshed *et al*.^86^ methods were used to determine GR, DHAR and MDHAR activities. A microplate reader (Synergy Mx, Biotek Instruments Inc., Winooski, VT, USA) were used for determination of all activities and scaled down for semi-high throughput to obtain linear time and protein concentration dependence.

### Statistical analysis

Data of four separate replications were reported as the mean ± SD. The data were statistically analyzed by analysis of variance (ANOVA) using Statistix 8 software. Duncan’s Multiple Range Test (DMRT) at 1% level of probability was used to compare the mean values. Microsoft Excel program was used to present the figures.

### Ethical statement

The lab and field experiment in this study was carried out following guidelines and recommendations of “Biosafety Guidelines of Bangladesh” published by Ministry of Environment and Forest, Government of the People’s Republic of Bangladesh (2005).

## Data Availability

Data used in this manuscript will be available to the public.
